# Doubly Fed Induction Generator Wind Turbines with Fuzzy Controller: A Survey

**DOI:** 10.1155/2014/252645

**Published:** 2014-06-15

**Authors:** J. S. Sathiyanarayanan, A. Senthil Kumar

**Affiliations:** ^1^Department of EEE, Arunai College of Engineering, Velu Nagar, Tiruvannamalai, Tamil Nadu 606603, India; ^2^Department of EEE, Velammal Engineering College, Chennai, Tamil Nadu 606603, India

## Abstract

Wind energy is one of the extraordinary sources of renewable energy due to its clean character and free availability. With the increasing wind power penetration, the wind farms are directly influencing the power systems. The majority of wind farms are using variable speed wind turbines equipped with doubly fed induction generators (DFIG) due to their advantages over other wind turbine generators (WTGs). Therefore, the analysis of wind power dynamics with the DFIG wind turbines has become a very important research issue, especially during transient faults. This paper presents fuzzy logic control of doubly fed induction generator (DFIG) wind turbine in a sample power system. Fuzzy logic controller is applied to rotor side converter for active power control and voltage regulation of wind turbine.

## 1. Introduction

Doubly fed induction generator (DFIG) is one of the most popular wind turbines which includes an induction generator with slip ring. Wind power is the most rapidly growing one since the 20th century due to its reproducible, resourceful, and pollution-free characteristics. Wind energy has a significant impact on dynamic behavior of power system during normal operations and transient faults with larger penetration in the grid. This brings new challenges in the stability issues and, therefore, the study of influence of wind power on power system transient stability has become a very important issue nowadays. Some years ago, the most common type of generators used in the wind energy conversion system (WECS) was the squirrel cage induction generator (SCIG), a fixed speed wind turbine generator (WTG) system, which has a number of drawbacks [1]. Most of the drawbacks can be avoided when variable speed WTGs are used. With the recent progress in modern power electronics, wind turbine with doubly fed induction generator (DFIG) has drawn increasing attention. In the DFIG, the induction generator is grid-connected at the stator terminals as well as at the rotor mains via a partially rated variable frequency AC/DC/AC converter (VFC). The VFC consists of a rotor-side converter (RSC) and a grid-side converter (GSC) connected back-to-back by a dc-link capacitor. The VFC takes a full control of the generator, like decoupled control of active and reactive power, faster dynamic response with low harmonic distortion, and so forth, handling only a very small fraction (25–30%) of the total power [2]. DFIG wind turbine also improves system efficiency with its optimal rotational speed, reduces noise and mechanical stresses, improves power quality, and compensates for torque and power pulsations [1]. When connected to the grid, the RSC of the DFIG may be blocked during a grid fault to protect it from overcurrent in the motor circuit [1, 2]. After the converter has been blocked, the wind turbine trips shortly. However, a few seconds after the fault has cleared, it automatically reconnects itself to the power network. The author in [3] proposed a feature of uninterrupted operation of a DFIG wind turbine during grid faults. The rotor circuit is first short-circuited by a crowbar circuit (an external resistor). The generator starts to absorb reactive power as it acts as a conventional induction generator. The operation of the wind turbine in producing active power continues and, to have a control over the reactive power and voltage, the GSC can be set. In order to prevent the wind turbine from fatal overspeeding, the pitch angle controller might be activated. The RSC will restart and the wind turbine will return to normal operation once the fault has been cleared and reestablish the voltage and frequency in the power network. However, for a weak power network with small power capacity, the GSC cannot provide sufficient reactive power and voltage support during a grid fault. The RSC will not restart, disconnecting the wind turbine from the power network. This introduces the risk of voltage instability. It is thus critical to maintain the voltage stability to maintain uninterrupted operation of the DFIG wind turbines.

As stated before, there is a global desire to institute large volume of wind power and flourish the share of energy consumption generated by the wind turbine. The interaction with the grid becomes extremely critical. Unlike conventional power plants, during and immediately following a grid failure, all the wind turbines will trip due to their inability to support the voltage and the frequency of the grid during the period. This might cause serious voltage recovery problems following a three-phase fault and begin to influence the overall power system stability [4]. For the smooth operation and optimal integration of large-scale application of wind energy, without any compromise to the system stability, it is thus essential for the turbines to stay connected to the grid, even in the case of a fault. Like conventional power plants, these renewable energy generators should be able to withstand and supply active and reactive power for frequency and voltage support immediately after the fault has been cleared to control and stabilize the power system following the disturbance [5]. The DFIG provides a huge possibility to improve network dynamic behavior by using the power electronic converters with sufficient control arrangements. Many different structure and control algorithms can be used for control of power converter. One of the most common control techniques is decoupled PI control of output active and reactive power to improve dynamic behavior of wind turbine. But due to uncertainty about the exact model and behavior of some parameters such as wind and wind turbine and also parameters values differences during operation because of temperature, events, or unpredictable wind speed, tuning of PI parameters is one of the main problems in this control method. Using fuzzy control, we can produce controller outputs more reliable because the effect of other parameters such as noise and events due to wide range of control region and online changing of the controller parameters can be considered. Moreover without the need for a detailed mathematical model of the system and just using the knowledge of the total operation and behavior of system, tuning of parameters can be done more easily.

## 2. DFIG Wind Turbine Model

**Figure 1:**
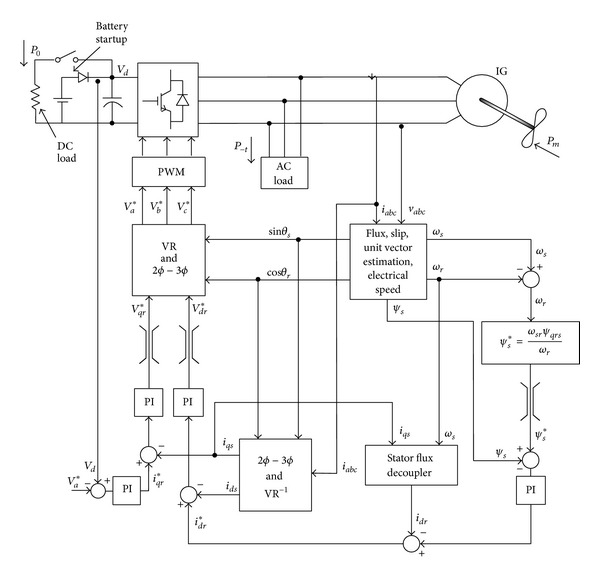
DFIG wind turbine.

Dynamic model of a DFIG wind turbine can be represented in terms of the equations of each of the subsystems, mainly the turbine, the drive train, the induction generator, and the control system. The detailed description of the dynamic modeling of DFIG wind turbine is out of the scope of this paper. Detailed information regarding this can be found in [6–10]. The block diagram of the basic DFIG is shown in Figure 1.

### 2.1. Wind Turbine Model

*C*_*P*_-*λ*-*β* curve demonstrates the characteristics of the aerodynamic model of a wind turbine. The mechanical torque (*T*_*m*_) extracted from the wind by the wind turbine can be expressed as [11]

(1) $$\begin{array}{c} T_{m}=\frac{\rho AC_{P}\left(\lambda ,\beta \right)V_{W}^{3}}{2\omega_{t}}, \end{array}$$

where *ρ* is the air density, *A* is the sweep area of the blades, *C*_*p*_ is the power coefficient, *λ* is the tip speed ratio, *β* is the pitch angle, *V*_*W*_ is the wind speed, and *ω*_*t*_ is the turbine rotor angular speed.

### 2.2. Drive Train Model

Conventionally, the rotor is treated as two lumped masses; that is, turbine mass and generator mass are connected together by a shaft with certain damping and stiffness coefficient values. After simplifications of neglecting the turbine and generator self-damping, shaft stiffness, and tensional oscillations, the mathematical equation for the mechanical part can be expressed as [12]

(2) $$\begin{array}{c} 2\left(H_{t}+H_{g}\right)\frac{d\omega_{g}}{dt}=T_{m}-T_{e}, \end{array}$$

where *H* is the inertia constant, *ω* is the rotor angular speed, and *T*_*e*_ is the electromagnetic torque; suffixes *t* and *g* denote the turbine and the generator, respectively.

### 2.3. Generator Model

A synchronously rotating *d*-*q* reference frame is chosen and generation convention is considered during modeling the induction generator. Park equations determine the values of *d*- and *q*-axis voltage value of stator and rotor [12], which in turn gives the values of electromagnetic torque and load flow (*P* and *Q*) determined by [13].

### 2.4. Control System Model

The control system generates pitch angle command signal, *β* for temporary reduction of wind turbine mechanical power by reducing *C*_*p*_ when the rotor speed is over the value that sustains power system stability. It also generates the voltage command signals *v*_*r*_ and *v*_gc_ for the rotor side converter (RSC) and grid side converter (GSC), respectively, in order to control the capacitor DC voltage and the reactive power or the voltage at the grid terminals.

### 2.5. Converter Protection Device

A protection device, namely, “Crowbar,” is used to save the rotor circuit and power electronic converter from high rotor transient current. Crowbar is external rotor impedance, coupled via the slip rings with the generator rotor instead of the converter. When the rotor current exceeds the current rating of the crowbar, it is triggered and the RSC is blocked. As long as the crowbar is triggered, the generator behaves as a conventional squirrel cage induction generator (SCIG) [3].

## 3. Evaluation of System Transient Stability

Power system transient stability is the capability of a power system to return to a stable operating point after the occurrence of a disturbance that changes its topology [14]. Examples of changes of the topology of a power system are

- tripping of a generator or a line,
- sudden change of a load, including a load trip,
- occurrence of a fault, that is, a short circuit.

**Figure 2:**
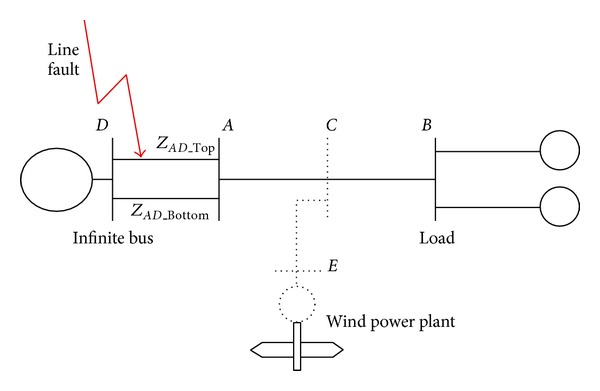
A test system suffering a short circuit fault.

To assess the system transient stability performance, the simplest and widely used test system is adopted in [6, 15–17] and a short circuit fault is simulated on one of the lines between Bus *A* and Bus *D* (Figure 2).

**Figure 3:**
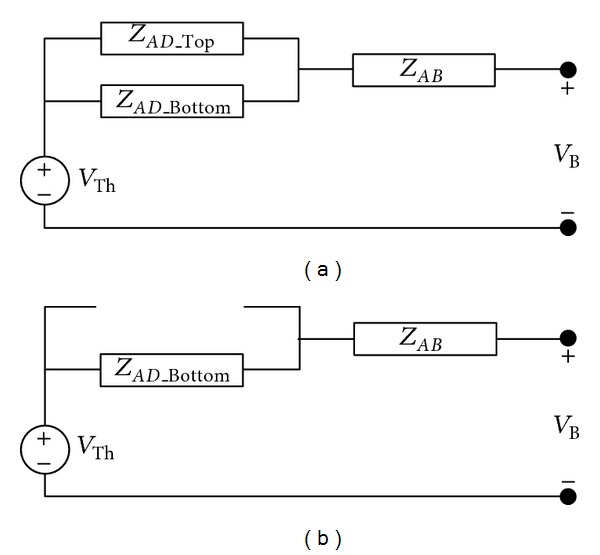
Thevenin equivalent circuit illustrating the fault process: (a) prefault operation and (b) postfault operation.

Thevenin equivalent circuit, seen from Bus *B*, is shown in Figure 3. This is generally called the driving point impedance at Bus *B* found from the *Z*_Bus_ matrix of the power system network. With reference to Figure 3, the Thevenin impedance before the fault

(3) $$\begin{array}{c} Z_{\text{Th}\_\text{Pre}F}=\left(Z_{AD\_\text{Top}}⊥Z_{AD\_\text{Bottom}}\right)+Z_{AB}, \end{array}$$

where *Z*_Th_Pre*F*_ is Thevenin impedance between Bus *D* and Bus *B* before the fault, *Z*_*AD*_Top_ and *Z*_*AD*_Bottom_ are the impedance of the lines between Buses *A* and *D*, and *Z*_*AB*_ = *Z*_*AC*_ + *Z*_*BC*_ is the impedance between Bus *A* and Bus *B*.

The Thevenin impedance after the fault is cleared is

(4) $$\begin{array}{c} \text{Th}\;\text{eF}\;AD\;\text{Bottom}\;AB\;Z=Z+\;Z\_\text{Pr}\_, \end{array}$$

where *Z*_Th_post*F*_ is Thevenin impedance between Bus *D* and Bus *B* after the removal of the fault line.

It is clear that *Z*_Th_Pre*F*_ is less than *Z*_Th_post*F*_, which weakens the system after fault. The weaker systems will have two types of impact: (1) the voltage drop across the Thevenin impedance will be larger and (2) the power transfer capability will be reduced. The transfer capability from one bus to another can be written as [17, 18]

(5) $$\begin{array}{c} P\;=\left(\frac{V_{1}*V_{2}}{X}\right)\sin\delta , \end{array}$$

where *V*_1_ is the magnitude of the bus voltage at the sending end, *V*_2_ is magnitude of the bus voltage at the receiving end, *X* is the reactance between Buses 1 and 2, and *δ* is the phase angle between Buses 1 and 2. When the voltage is recovering right after the fault, the power transfer capability is decreased proportionally to the amount of voltage reduced at Bus 1 or Bus 2 or both. The voltage regulator will recover the voltages at Bus 1 and Bus 2 after some time to restore the power transfer capability. However, like the case presented here, if the network configuration changes after the fault the reactance *X* after the fault increases significantly thus changing the power transfer capability.

**Figure 4:**
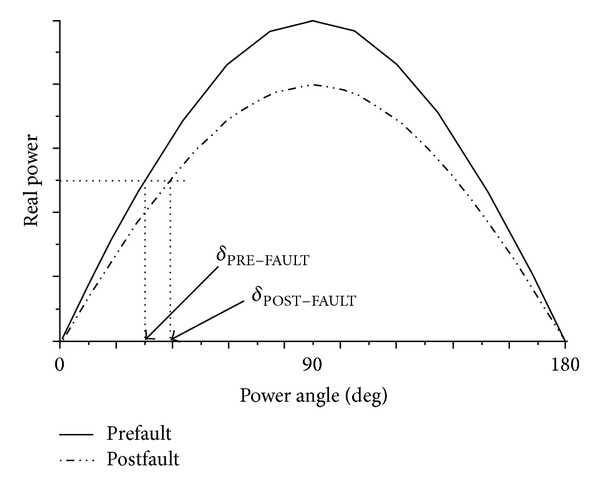
Power transfer between two buses.

The changes in the power-angle characteristic, representing the power transfer between the two buses, are represented in Figure 4. The prefault capability curve shrinks to the postfault curve during the process. Due to the constant load transfer from the source to the sink, the power angle  *δ*  increase as the grid weakens (*δ*_POST-FAULT_ > *δ*_PRE-FAULT_). The operating *δ* may be reasonably small at the prefault condition. During the postfault condition, the operating *δ* angle increases closer to 90°, making the power system more prone to instability. The synchronous machines at two ends of the line may lose synchronism with respect to each other after a system fault, if the operating  *δ*  is initially large and close to the stability limit.

## 4. Transient Phenomena with DFIG Wind Turbines

This section gives a brief highlight of the transient phenomena with grid connected DFIG wind turbines. Detailed descriptions can be found in [6, 11, 19].

### 4.1. During the Fault

In the fault instant, due to short circuit, the voltage at the DFIG generator terminal drops. This has got several consequences.

- Generator rotor and stator flux decrease resulting in generator demagnetizing process. The consequent result is the reduction in the electromagnetic torque and active power of the generator. Mechanical torque gets higher as compared to the electromagnetic torque and therefore the generator starts to accelerate.
- High current transients appear in the stator and rotor windings. Crowbar is triggered to bypass this high current away from the RSC to prevent the converters from any kind of destructions.
- The GSC is not able to transfer the whole power from the rotor through the converter further to the grid. As a result, the additional energy goes into charging the dc-bus capacitor rapidly.
- Due to decouple control of mechanical rotor frequency from the grid frequency by means of power electronic converters, a part of potential energy stored in the rotating mass of the shaft of DFIG wind turbine cannot be supplied to the system. This fact resists the DFIG wind turbine from restoration of grid frequency [14].
- It causes exciting oscillations in the rotating mass of the shaft as well, which is severe with higher time constant and higher damping time due to manufacturing WTG with softer shaft [20].

### 4.2. After Clearance of the Fault

When the fault is cleared, the voltage cannot recover completely immediately because RSC cannot provide necessary reactive power to the generator for its magnetization process as long as it is blocked due to triggering of crowbar. The generator thus needs to absorb reactive power from the grid and this action delays the recovering process of the grid voltage. GSC successfully controls the dc-voltage back to its nominal value. When the grid voltage recovers over a certain value, the crowbar is removed. From this moment, the voltage recovers completely, the generator currents and voltages start to converge to their prefault values, and the RSC retains its control over the active and reactive power.

### 4.3. Factors Influencing Transient Stability

Fault duration, strength of grid coupling, or other mechanical properties play only a minor role in fault responses if fault ride through operation continues. Only protection system setting has a major impact on fault responses and hence transient stability, only when WTG disconnection is induced by voltage or frequency deviations. If reconnection time and/or ramping time (time it takes before WTG has returned to its normal operating regime) are very long, the generated electrical power goes much lower than the extracted mechanical power (which is hardly reduced) and an unbalance between mechanical and electrical torque results both before reconnection time and during ramping time. This is how transient stability is prolonged [4].

## 5. Fault Ride through Operation

DFIG wind turbines are required to be disconnected from the grid to prevent from going into system instability due to the vulnerable occurrences like high transient current flow, oscillations excited to rotating mass of the drive train, rotor over speed, and dip in grid voltage due to the fault. But with more wind power penetration in the grid, WTGs are having more influences on the overall power system stability and hence “fault ride through operation” (which means remaining connected in the grid following a fault) of these WTGs is listed as requirement in the grid codes of countries with high level of wind power penetration [21].

To sustain fault ride through operation of DFIG wind turbines, several control and power electronic devices have been implemented to mitigate the abovementioned vulnerable factors immediately after the fault so that the power system stability is retained within a few seconds after its clearance. These are discussed as follows.

### 5.1. Converter Controller

Installation of crowbar in the system increases the cost and hinders its reliability. Moreover, triggering of a crowbar causes RSC blocking, which results in having generator to operate as SCIG. This often forces the disconnection of WTG, which is a complete violation of grid code requirement. Limitation of rotor current has been achieved without any disconnection by converter control method. Several advanced control principles have been reported: (i) eliminating current and voltage unbalances by supplying negative sequence current [22–24], (ii) making the components of rotor current oppose the undesired dc and negative sequence components in the stator flux linkage, and (iii) making arrangements to feedback the measured stator currents as the set point for the current controller of the RSC [25].

### 5.2. Damping Controller

Oscillation excited to rotating mass of the drive train followed by a grid fault may prolong system stability. PI damping controller can damp “fast generator speed oscillations” very effectively. It produces active power reference signal for the RSC control based on the deviation of generator rotor speed from its reference. The speed reference is defined by the optimal speed curve at the incoming wind. The damping controller needs proper tuning. Insufficient tuning may lead to self-excitation of the drive train system and risk of unnecessary tripping as protection against vibration in the mechanical construction [26].

### 5.3. Dynamic Reactive Compensator

During voltage recovery process following a transient fault, GSC cannot provide necessary reactive power due to having converter with small capacity. That is why the generator absorbs reactive power from the grid and results in voltage and frequency instability. Such instability problems can be solved by dynamic compensation of generator's reactive power demand by the following two methods: (i) using shunt flexible AC transmission system (FACTS) devices, such as the static VAR compensator (SVC) and the static synchronous compensator (STATCOM), to generate a set of balanced three-phased sinusoidal voltages at the fundamental frequency with rapidly controllable amplitude and phase angle to drive PWM converter with dc-link capacitor and transport necessary reactive power in the grid [5, 27, 28], and (ii) reconfiguring RSC in parallel with GSC during fault so that both feed a good amount of reactive current, which is again intensified by transient control mode (TCM) [29]. This is how a rapid regulation of the grid voltage is maintained to enhance the capability of the DFIG wind turbine to ride through the fault.

### 5.4. Pitch Angle Controller

At a short circuit fault, the grid voltage and then the electromagnetic torque are significantly reduced, which lead to wind turbine acceleration. The pitch angle controller therefore prevents the rotor overspeed and hence helps reestablishing the voltage at the WTG terminal by aerodynamic power shedding by the generation of pitch angle. It should be possible to reduce the power production of the wind turbine from any arbitrary operational point to below 20% of the rated power in less than 2 s [19]. Faster pitch rate of change can support better the network voltage stability by reducing aerodynamic power faster. Pitch angle controller damps “slow frequency oscillations of generator speed” as well.

## 6. Indirect Current Mode Control

This section analyzes the closed-loop behaviour of the DFIG system using indirect CMC methods. Two current regulators for the rotor-side converter are considered: the conventional regulator and a regulator intended for unbalanced grid faults. The same steps are followed in both analyses: the open-loop model of the DFIG system is first formulated; second, the current regulator is written; and, third, by combining the open-loop model and the control model, the closed-loop dynamic description of the rotor-side system is derived.

### 6.1. Conventional Current Regulator

The open-loop model of the DFIG system is represented, as usual, in the *d* − *q* reference frame. A synchronously rotating reference frame is used with the direct axis oriented along the stator flux position. In this way, the reference frame is rotating with the same speed as the stator voltage. Thus, the stator and rotor voltages can be expressed as follows:

(6) $$\begin{array}{c} V_{ds}=R_{s}i_{ds}+\frac{d\psi_{ds}}{dt}-\omega_{s}\psi_{qs}\\ V_{qs}=R_{s}i_{qs}+\frac{d\psi_{qs}}{dt}+\omega_{s}\psi_{ds}\\ V_{dr}=R_{r}i_{dr}+\frac{d\psi_{dr}}{dt}-\left(\omega_{s}-\omega_{r}\right)\psi_{qr}\\ V_{qr}=R_{r}i_{qr}+\frac{d\psi_{qr}}{dt}+\left(\omega_{s}-\omega_{r}\right)\psi_{dr} \end{array}$$

with

(7) $$\begin{array}{c} \psi_{ds}=L_{s}i_{ds}+L_{\text{midr}}\\ \psi_{qs}=L_{s}i_{qs}+L_{\text{miqr}}\\ \psi_{dr}=L_{r}i_{dr}+L_{\text{mids}}\\ \psi_{qr}=L_{r}i_{qr}+L_{\text{miqs}}. \end{array}$$

The inputs of the open-loop model are voltages and angular frequencies, and the outputs are currents. The stator voltage is given by the grid voltage, while the rotor voltage is supplied by the rotor-side converter. It is assumed in this study that the converters are sufficiently fast, so that they can exactly track the reference voltage. In this case, the rotor voltages are given by the rotor-side controller:

(8) $$\begin{array}{c} V_{dr}=V_{dr}^{*}\\ V_{qr}=V_{qr}^{*}. \end{array}$$

**Figure 5:**
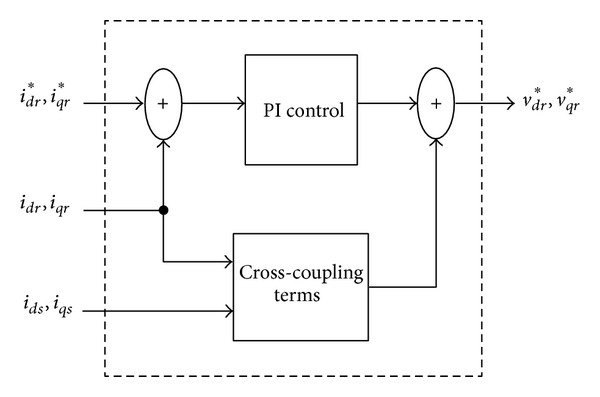
Conventional (indirect) current regulator.

Figure 5 shows the conventional current regulator for the rotor side converter. The regulator combines a proportional-integral (PI) controller with cross-coupling:

(9) $$\begin{array}{c} V_{dr}^{*}=R_{r}i_{dr}+V_{dr}^{\text{pi}}-\left(\omega_{s}-\omega_{r}\right)\psi_{qr}\\ V_{qr}^{*}=R_{r}i_{qr}+V_{qr}^{\text{pi}}+\left(\omega_{s}-\omega_{r}\right)\psi_{dr}. \end{array}$$

## 7. Fuzzy Control System

The control system is based on fuzzy logic. This type of control, approaching the human reasoning that makes use of the tolerance, uncertainty, imprecision, and fuzziness in the decision-making process, manages to offer a very satisfactory performance, without the need of a detailed mathematical model of the system, just by incorporating the experts' knowledge into fuzzy rules. In addition, it has inherent abilities to deal with imprecise or noisy data; thus, it is able to extend its control capability even to those operating conditions where linear control techniques fail (i.e., large parameter variations). This system has four main parts. First, using input membership functions, inputs are fuzzified, then, based on rule bases and inference system, outputs are produced, and finally the fuzzy outputs are defuzzified and applied to the main control system. Error of inputs from their references and error deviations in any time interval are chosen as inputs. In this paper, these parts as illustrated in Figure 5 are simulated in MATLAB. Fuzzy logic Control units in MATLAB are linked with main system in EMTDC software; therefore, in any simulation time interval, both software solutions work simultaneously. Inputs are sent to MATLAB and output fuzzy controllers are produced there and sent to EMTDC software for the main system. The output of fuzzy controller is the value that should be added to the prior output to produce new reference output.

**Figure 6:**
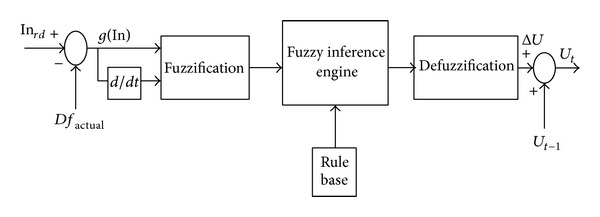
Block diagram of fuzzy controller.

Figure 6 shows the block diagram of rotor side converter to which fuzzy controllers are applied. The main objectives of this part are active power control and voltage regulation of DFIG wind turbine using output reactive power control. As illustrated in Figure 6 rotor-side converter manages to follow reference active (*P*_ref_) power and voltage (*V*_ref_) separately using fuzzy controllers, hysteresis current controller converter, and vector control algorithm [30]. Based on inputs of fuzzy controller are errors in active and reactive power or voltage and the rate of changes in errors in any time interval.

After the production of reference *d*-axis and *q*-axis rotor currents, they converted to *a*-*b*-*c* reference frame using flux angle, rotor angle, and finally slip angle calculation and Concordia and Park transformation matrix. Then they are applied to a hysteresis current controller to be compared with actual currents and produce switching time intervals of converter.

## 8. Conclusion

With the increasing wind power penetration, the wind farms (mostly equipped with increasing number of DFIG wind turbines) are directly influencing the power systems. Therefore, grid code of different countries has enlisted “fault ride through operation” of WTGs as one of the major requirements to sustain transient stability for reliable operation of power system with uninterrupted power generation and supply. It shows that the study of transient dynamics with DFIG wind turbines with its current research stand and future research options is a very important issue. Generally, DFIG wind turbine suffers from high transient current, rotor overspeed, oscillations in the rotating mass of the shaft, grid voltage, and frequency excursions while subjected to a transient fault. Research efforts throughout the world result in various advanced scientific inventions of devices like advanced converter controller, damping controller, pitch angle controller, and dynamic reactive compensator. These devices help mitigate vulnerable problems addressed by transient faults. Our exploration is to make sustenance of “fault ride through operation” of DFIG wind turbines as a means of enhancing transient stability of power systems. Extensive studies have been carried out on transient phenomena, factors influencing transient phenomena, and the impact of faults on both DFIG wind turbine and the grid. But, to our knowledge, most of them carried out qualitative research on transient stability. Most of the papers in the literature analyzed transient dynamics and stability followed by symmetrical faults on a system with constant loads. Fuzzy controllers, in contrast to the conventional PI controllers, can take care of the nonlinearity in the control law and hence are known to have better performance than PI under variable operating conditions. Moreover, the TS-fuzzy is better than the mamdani type fuzzy controllers in terms of the number of fuzzy sets for the input fuzzification, number of rules used, and the number of coefficients to be optimized. These studies may contribute to more accurate simulation results for finding out better strategies for smooth, reliable, and uninterrupted operation of power networks with the future penetration of wind energy systems.
